# Fatal Human Rabies due to Duvenhage Virus from a Bat in Kenya: Failure of Treatment with Coma-Induction, Ketamine, and Antiviral Drugs

**DOI:** 10.1371/journal.pntd.0000428

**Published:** 2009-07-28

**Authors:** Pieter-Paul A. M. van Thiel, Rob M. A. de Bie, Filip Eftimov, Robert Tepaske, Hans L. Zaaijer, Gerard J. J. van Doornum, Martin Schutten, Albert D. M. E. Osterhaus, Charles B. L. M. Majoie, Eleonora Aronica, Christine Fehlner-Gardiner, Alex I. Wandeler, Piet A. Kager

**Affiliations:** 1 Department of Infectious Diseases, Tropical Medicine and AIDS, Academic Medical Center, University of Amsterdam, Amsterdam, The Netherlands; 2 Department of Neurology, Academic Medical Center, University of Amsterdam, Amsterdam, The Netherlands; 3 Department of Intensive Care, Academic Medical Center, University of Amsterdam, Amsterdam, The Netherlands; 4 Department of Clinical Virology, Academic Medical Center, University of Amsterdam, Amsterdam, The Netherlands; 5 Department of Virology, Erasmus Medical Centre, Rotterdam, The Netherlands; 6 Department of Radiology, Academic Medical Center, University of Amsterdam, Amsterdam, The Netherlands; 7 Department of Pathology, Academic Medical Center, University of Amsterdam, Amsterdam, The Netherlands; 8 Centre of Expertise for Rabies, Canadian Food Inspection Agency, Ottawa Laboratory (Fallowfield), Ottawa, Ontario, Canada; New York University School of Medicine, United States of America

## Introduction

Rabies, encephalitis caused by lyssaviruses, was considered universally fatal until a young, unvaccinated patient with bat rabies survived after a new therapeutic approach [1]. The long-term favourable outcome of this patient suggested that successful interventions may be possible [2]. However, 11 patients, subsequently treated in a similar way, have not survived (reviewed by Wilde et al. [3]). Of six of these patients, information is published [4]–[8]; the other five are mentioned in the review [3].

We report on a patient with rabies due to Duvenhage virus from a bat in Kenya. A very brief description of this case without clinical details and without results of virological and pathological investigations was reported in *Eurosurveillance* [7]. This report was included in the review of Wilde et al. [3]. We now present this patient in more detail and with results of virological and pathological investigations. We received written consent for publication of this case report from the patient's husband.

## Case Report

A bat flew against the face of a 34-year-old Dutch woman, a medical doctor, on a campsite in Tsavo West National Park, Kenya. She swept the animal away and noticed two small superficial bleeding wounds on the right side of the nose. The wounds were washed with water and soap, and cleaned with alcohol swabs. The park wardens and the personnel at the nearby health facility assured her that locally rabies was only known to be carried by dogs and cats. She was not referred for postexposure prophylaxis.

Fourteen days after her return to The Netherlands, 23 days after the incident with the bat, she complained of malaise, dizziness, muscle aches, and headache. She had no fever. Two days later, she experienced difficulty with speech and swallowing. There was hypaesthesia of both cheeks and she felt unsteady. She vomited once. She was admitted in isolation the next day, day 4 of complaints. Her past medical history was unremarkable and she did not take medication.

On examination she was anxious, but alert and well oriented. Her temperature and other vital parameters were normal. There was no hypersalivation, hydrophobia, or aerophobia, and no difficulty swallowing. The wounds on the nose were resolved; there was no itch or pain. There was a slight dysarthria and she spoke in a staccato way. Apart from the hypaesthesia of both cheeks, further neurological examination was unremarkable. The deep tendon reflexes were normal. On advice of the National Coordination Center for Infectious Diseases (NCCID), she received human anti-rabies immunoglobulin (HRIG), 20 IU/kg im, divided over both quadriceps muscles, and human diploid rabies vaccine (Mérieux) im (days 1, 4, 8, and 15 of admission). Other infections, such as Rift Valley fever, West Nile fever, lymphocytic choriomeningitis, yellow fever, enterovirus 71 and *Listeria monocytogenes* infection, malaria, and trypanosomiasis were considered and ruled out by appropriate tests (immunofluorescence or enzyme-linked immunosorbent assay, culture of blood and cerebrospinal fluid (CSF) and microscopy of blood slides and CSF, PCR for enterovirus, inclusive of enterovirus 71). Neurological considerations included infection by neurotropic viruses, acute disseminated encephalomyelitis, and, initially, anxiety disorder. On day 2 of admission, her temperature was 38.5°C, the dysarthria had worsened, and she complained of difficulty with swallowing. She was agitated and she had a high frequency postural tremor of her hands.

**Figure 1:**
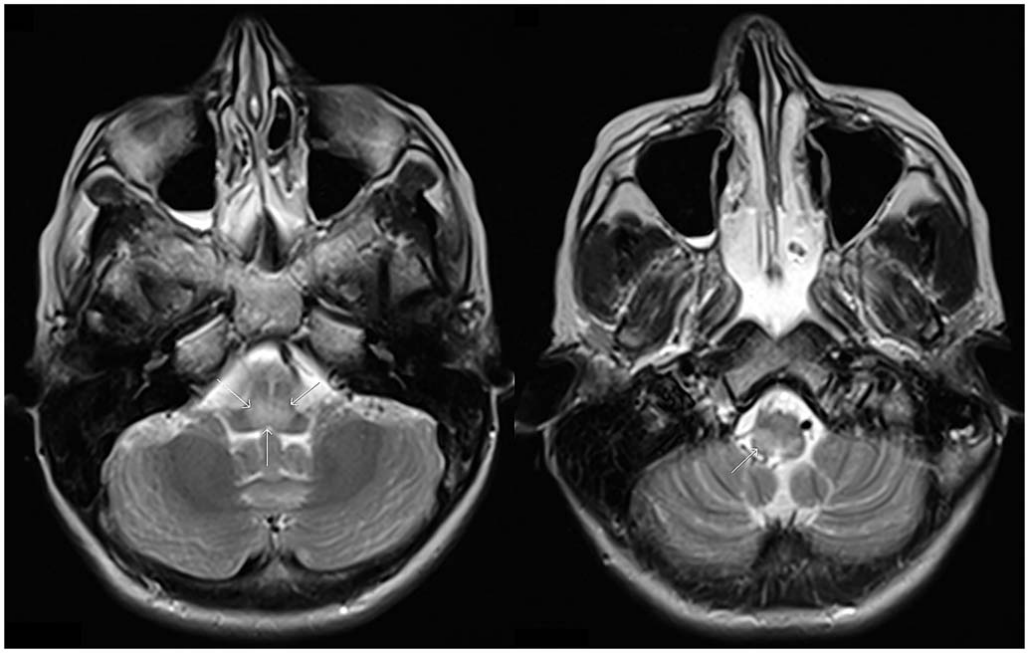
MRI brain on day 6 of admission. Axial T2-weighted images in a patient with rabies due to Duvenhage virus infection, at day 6 after admission; hyperintense signal in the posterior part of the medulla oblongata (arrow).

Serum, CSF, and a nuchal biopsy were sent to the rabies reference laboratory, Department of Virology, Erasmus Medical Centre, Rotterdam. The CSF showed 12 white blood cells (lymphocytes)/3 mm^3^ (*n*<5), no red blood cells, a protein level of 0.23 g/l (*n*<0.5) and a glucose level of 3.0 mmol/l (blood glucose 4.9 mmol/l). During the following days her dysarthria increased. She complained of weakness of arms and legs and double vision. She developed hypersalivation and became hyperreactive to noises. Her temperature was variable with bouts of fever, up to 39.2°C. On day 6 of admission, the condition acutely worsened; she had an epileptic seizure and aspirated, and oxygenation dropped due to respiratory insufficiency. She was intubated and admitted to the Intensive Care Unit (ICU). The CSF now showed 64 white blood cells (no differential count performed) and 25 red blood cells/3 mm^3^, a protein level of 0.72 g/l, and an elevated IgM index of 0.35. An MRI showed an area of increased signal intensity on T2-weighted images in the posterior part of the medulla oblongata and pons (Figure 1). The next day she was drowsy and there was nuchal rigidity. She opened her eyes in response to voices and she squeezed with her left hand and moved her legs on command. She had horizontal and downward gaze palsies. The deep tendon reflexes of the arms were absent, while the leg reflexes were normal.

Rabies seemed the probable cause of the encephalitis, but the pharyngo-cervical-brachial variant of Guillain-Barré syndrome (GBS) was also considered. No rabies-specific antibodies had been detected in serum collected before vaccination or in serum or CSF from day 2 of admission, and *Lyssavirus*-specific RT-PCR was negative in those specimens. The nuchal biopsy, obtained on day 2 of admission, showed a weak band of correct size on agarose gel in nested RT-PCR in only one of the quadruplicate samples and therefore required confirmation by cloning and sequencing [9] (Box 1). Treatment with intravenous immunoglobulin 0.4 g/kg/d for 5 days as for GBS and with acyclovir 10 mg/kg three times per day iv for 5 days as for herpes simplex encephalitis was started (PCR for herpes simplex virus and varicella zoster virus in serum and CSF proved negative). Before rabies was confirmed, the treatment protocol used for the rabies patient who survived [1] was started on day 7 of admission, after information was provided to her husband and family and with their consent. Treatment consisted of induction of coma with pentobarbital 4 mg/kg/h, midazolam 5 mg/h, and ketamine 100 mg/h; the EEG was continuously monitored and pentobarbital and midazolam were titrated to burst suppression, meaning to a maximum of four bursts per minute. Administration of the antiviral amantadine 100 mg twice per day per nasogastric tube was started the next day (day 8 of admission) and ribavirin iv, 1 g four times per day, was added from day 12 onwards. Tetrahydrobiopterin and co-enzyme Q10 were given from day 15. A complicating pneumonia was treated with cefotaxim 1 g iv, four times per day for 5 days. Routine ICU procedures of prophylactic administration of low molecular weight heparin, regulation of blood glucose level by continuous administration of glucose 5% and insulin, and selective decontamination of digestive tract (SDD) [10] were applied. SDD consists of a four times daily application to the buccal cavity of 0.5 g of an oral paste containing 2% polymyxin E, 2% tobramycin, and 2% amphotericin B, and once daily administration of 100 mg polymyxin E, 80 mg tobramycin, and 500 mg amphotericin B through a nasogastric tube.

**Box 1. Virological Investigations of a Patient with Duvenhage Virus Infection**

Clinical samples

Serum, d2 antibody and PCR neg, d7 antibody neg;

CSF, d2 antibody and PCR neg, d 6 and d 28 Lyssa PCR neg;

Nuchal skin biopsy, d2 PCR pos, see text, IF rabies neg, d10 PCR neg (repeated because of delay with first biopsy);

Saliva, d7 Lyssa PCR pos (reported d 13), d10 neg;

Cornea smear, d7 IF neg.

(Note: results of tests performed.)

On day 11 of admission the presence of Duvenhage virus in the nuchal skin biopsy specimen from the 2nd day of admission was confirmed. This may seem like a long delay, but only one of the two agarose gels tested by PCR showed a faint band, and the repeated test in duplicate was negative in both tests. The amplicon had to be cloned as direct sequencing was not successful. Details of virological investigations are presented in Box 1.

Blood pressure, heart rhythm, kidney function, and diuresis were regulated by fluids and low dose noradrenaline; a supervening lung infection with *Staphylococcus aureus* was treated with flucloxacillin iv, 1 g, four times per day for 7 days. On admission to the ICU, the temperature was elevated up to 40.1°C, but after administration of acetaminophen suppositories (1,000 mg, four times per day, in total given for 3 days) the temperature was normal 24 hours later and remained normal thereafter without further measures. Diabetes insipidus, first apparent on day 12 of admission, day 7 of ICU, was regulated with iv fluids to which desmopressin was added on days 13, 16, and 17. Pentobarbital was stopped after 5 days when no bursts were seen on the EEG and midazolam was stopped after 7 days of administration. Twenty-four hours later, 3 days after stopping pentobarbital and while there was no pentobarbital detectable in the serum, the EEG showed a very low voltage background pattern.

**Figure 2:**
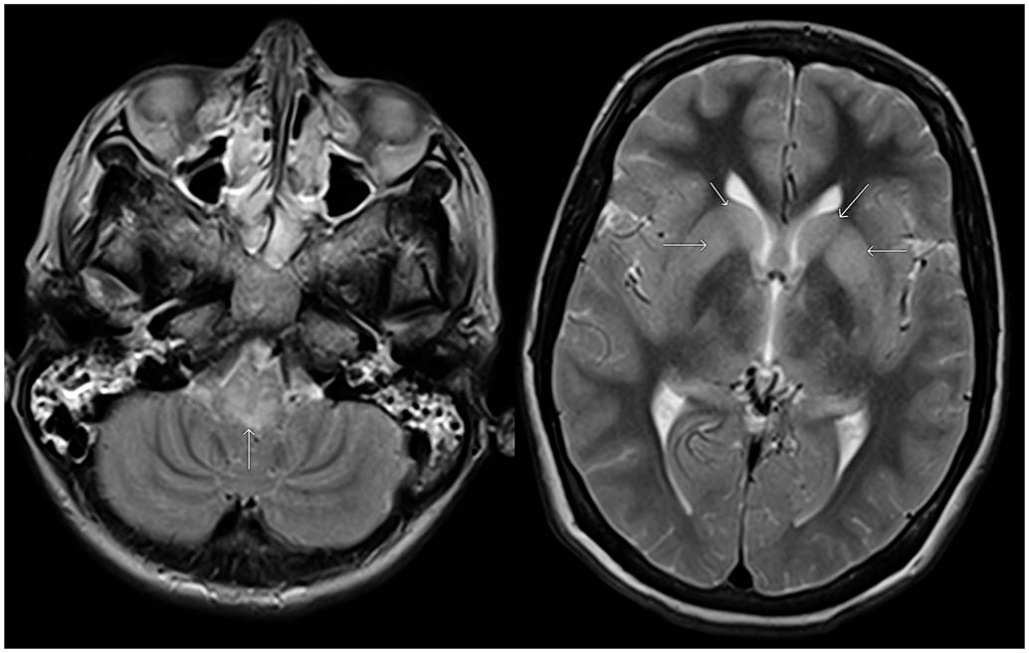
MRI brain on day 16 of admission (4 days before death). Axial T-2 weighted images in a patient with rabies due to Duvenhage virus infection, at day 17 after admission show increased signal in the posterior part of the medulla oblongata (arrow) and in the basal ganglia (arrows).

MRI abnormalities of the medulla oblongata had increased and now extended to mesencephalon and both dentate nuclei in the cerebellum (Figure 2). Also, there was diffuse swelling of both hemispheres. Magnetic resonance angiography did not reveal vascular abnormalities. There was no response to pain or acoustic stimuli after withdrawal of ketamine. Brainstem reflexes and deep tendon reflexes were absent. Introduction of a tube to clean the airways did not result in a response of coughing and there was no spontaneous respiration. In agreement with the wishes of the family it was decided to stop ventilatory support. She died on day 20 of admission, 45 days after the incident with the bat.

**Figure 3:**
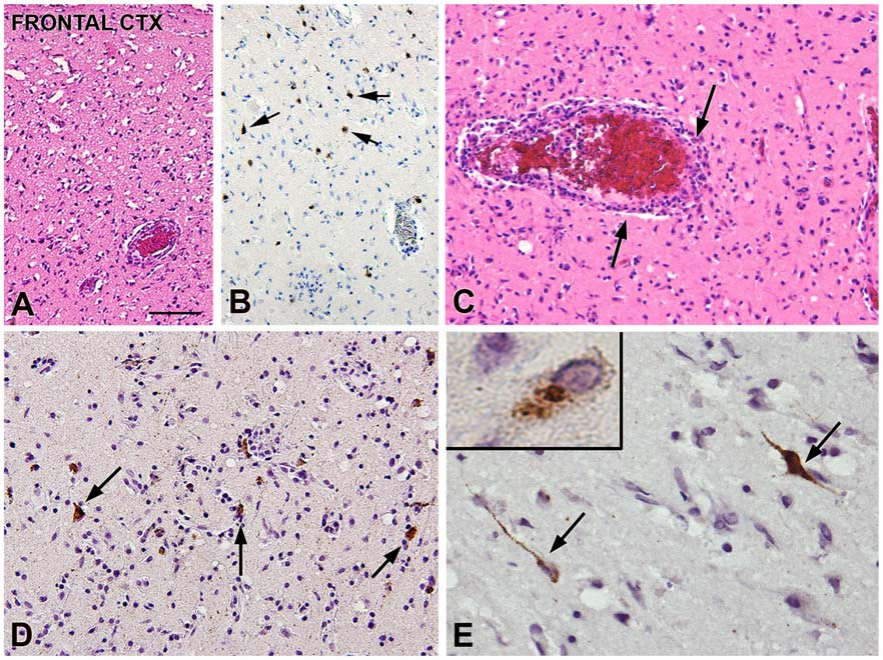
Histopathology rabies encephalitis due to Duvenhage virus. Rabies encephalitis (lyssavirus; genotype 4) brain pathology: cerebral cortex and anti-RNP Immunohistochemistry. (A) (HE staining): frontal cortex: extensive neuropil vacuolization and neuronal cell loss. (B) Few residual cortical neurons (arrows). (C) (HE staining): perivascular lymphocytic inflammatory infiltrates (arrows). (D and E) Anti-RNP immunoreactivity within the frontal cortex. Several neuronal cells show immunopositivity (arrows and insert in [E]) with multiple antigenic masses present in the neuronal cytoplasm, as well as in dendrites and axon. Scale bar: (A, B) 100 µm; (C, D) 60 µm; (E) 40 µm; insert in (E): 12 µm.

At postmortem examination no gross abnormalities were found, apart from the pneumonia. The brain was swollen and weighed 1,360 g. Histopathology of the cerebral cortex (Figure 3A and 3B) showed extensive neuropil vacuolization, neuronal cell loss, astrogliosis, and widespread inflammatory changes consisting of macrophages, activated microglia, and lymphocytic infiltrates. Severe neuronal cell loss was observed in the hippocampus, midbrain, pons, medulla oblongata, and cerebellum. Immunohistochemistry using polyclonal rabbit anti-rabies nucleoprotein antibody showed rabies virus antigen in the frontal and temporal cortex, as well as in the hippocampus and entorhinal cortex (Figure 3C and 3D). Rabies virus antigen was not detected in other regions of the brain nor in adrenal glands, salivary gland, pancreas, thyroid, heart, or gastrointestinal tract.

## Materials and Methods

### Antibody Detection

The Platelia Rabies II Kit (Bio-Rad, Marnes-La-Coquette, France) was used to detect and titrate IgG anti-rabies virus glycoprotein in serum or plasma. The test was also used to detect specific antibody in CSF.

### Antigen Detection Using Immunofluorescence

*Lyssavirus* antigen was detected by the immunofluorescence examination using Bio-Rad Rabies Anti-nucleocapsid Conjugate (Bio-Rad, Marnes-La-Coquette, France). Nuchal skin biopsies and corneal smears were examined by the immunofluorescence assay to detect the presence of rabies virus antigen.

### Cell Culture

**Figure 4:**
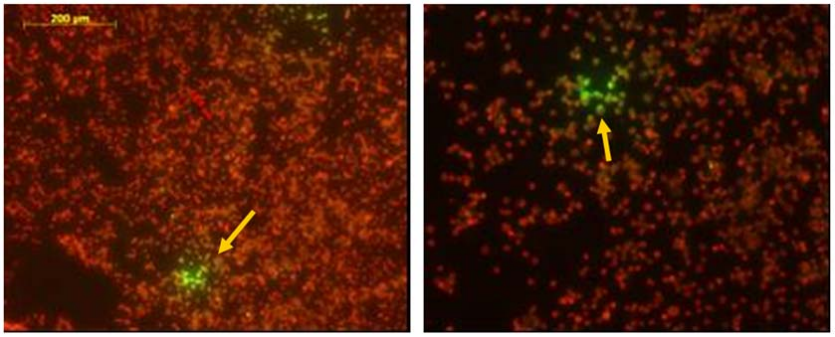
Immunofluorescence tests with antirabies nucleocapside conjugate. Immunofluorescence of murine neuroblastoma cells using antirabies nucleocapside conjugate (Bio-Rad). The arrow points to cells with positive green immunofluorescence. The right picture is a magnification of the left with the scale bar.

Cell culture of the various clinical samples was carried out by inoculation of murine neuroblastoma cells and BHK-21 cells propagated in 24-well microtiter plates. CSF was tested without processing, and saliva was made up to a volume of 2 mL with virus transport medium. The other specimens were ground with virus transport medium up to a volume of 5 mL. After centrifugation a volume of 200 µL was added to each well. Immunofluorescence tests with anti-rabies nucleocapside conjugate (Bio-Rad) showed immunopositivity as presented in Figure 4.

### Heminested PCR and Sequence Analysis

A rabies-specific diagnostic RT-PCR was performed as previously described [9]. In short, nucleic acid was extracted using the MAgNA Pure LC (Roche Diagnostics, Penzberg, Germany) with the Total Nucleic Acid Isolation kit. Nested RT-PCR with primers from the nucleoprotein encoding region was carried out with read-out on agarose gel. Positive bands were isolated from the agarose gel using the Qiagen Quick Gel Extraction Kit (Qiagen, Hilden, Germany) according to the manufacturer's instructions. From the gel, extracted RT-PCR amplicons were sequenced with the ABI BigDye Terminator version 3.1 Cycle Sequencing Kit (Applied Biosystems, Nieuwerkerk a/d IJssel, The Netherlands) on an ABI 3100. Sequences were generated with the Lasergene version 7 software package (DNASTAR, Madison, United States) and blasted at the NCBI database using a blastn algorithm in the nr database.

**Figure 5:**
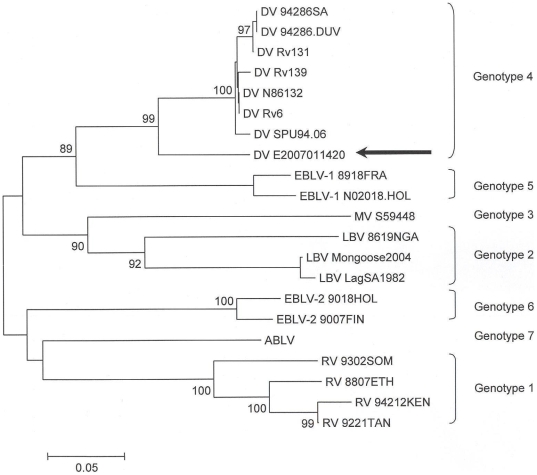
Lyssa virus phylogenetic tree with presented rabies case embedded. Phylogenetic tree of the presented rabies case (sequence arrowed) and representative *Lyssavirus* genomes. Mega 3.1 tree (neighbour joining, Kimura 2–parameter, 1,000 bootstrap values) based on a 367–base pair fragment in the nucleoprotein region. Bootstrap percentages higher than 75% are indicated. Genotype 1  =  rabies virus (RV); Genotype 2  =  Lagos bat virus (LBV); Genotype 3  =  Mokola virus (MV); Genotype 4  =  Duvenhage virus (DV); Genotype 5  =  European bat lyssavirus (EBLV) 1a and 1b; Genotype 6  =  European bat lyssavirus (EBVL) 2a and 2b; Genotype 7  =  Australian bat lyssavirus (ABLV). Present virus isolate in Genotype 4: DV E2007011420 (arrowed).

A 367–base pair fragment in the nucleoprotein region was sequenced and phylogenetic analysis was performed using Mega 3.1 (neighbour joining, Kimura 2–parameter, 1,000 bootstrap values) [11] (Figure 5).

It should be noted that a Duvenhage strain had not been present in our laboratory.

### Mouse Inoculation Test

Newborn NIH outbred mice (RIVM, Bilthoven, The Netherlands) were inoculated intracerebrally with the various clinical samples (submaxillary salivary gland, tectum, cerebellum, and hippocampus). The preparation of the specimens was the same as for the cell culture. The specimens were homogenized and a volume of 15 µL was introduced into the central part of the upper parietal area of the calvaria. The inoculated mice were observed daily for a maximum of 28 days; the inoculated mice did not show symptoms of infection. Rabies virus antigen could not be detected by immunofluorescence of the mouse brains; furthermore, the brains were also negative by PCR.

### Further Serological Assays

Antibodies against lymphocytic choriomeningitis virus, Rift Valley virus, West Nile virus, and yellow fever virus were determined by immunofluorescence assays or enzyme-linked immunosorbent assays.

### Tissue Preparation

Tissue was fixed in 10% buffered formalin and embedded in paraffin. Paraffin-embedded tissue was sectioned at 6 µm, mounted on organosilane-coated slides (Sigma-Aldrich, St. Louis, Missouri, United States), and used for hematoxylin and eosin (H&E) staining. Apart from H&E staining, representative sections of all brain specimens were processed for conventional histochemical staining (Bielschowsky, Palmgren silver stain, Klüver-Barrera, and Nissl) and immunohistochemistry.

### Immunohistochemistry

Several blocks of different brain regions (frontal-, temporal-, occipital-, motor-cortex; hippocampus, entorhinal cortex; mesencephalon, pons, medulla oblongata, cervical cord, and cerebellum) were used for immunohistochemical analysis with neuronal nuclear protein (NeuN; mouse clone MAB377; Chemicon, Temecula, California, United States; 1∶2000).

For the detection of the virus, polyclonal rabbit anti-rabies-nucleoprotein antibody (RNP; 1∶500, Centre of Expertise for Rabies, Ottawa, Canada) was used. The immunostaining with this anti-rabies antibody was also performed on material from adrenal gland, salivary gland, pancreas, thyroid, heart, and gastrointestinal tract.

Immunohistochemistry was carried out as previously described [12]. Briefly, paraffin-embedded sections were deparaffinized, re-hydrated, and incubated for 20 min in 0.3% H_2_O_2_ diluted in methanol. Antigen retrieval was performed by incubation for 10 min at 121°C in citrate buffer (0.01 M, pH 6.0) or for the anti-RNP with proteinase K pretreatment (5 mg/ml for 5 minutes at 20°C; Boehringer Mannheim, Mannheim, Germany). After incubation with the primary antibodies overnight at 4°C, ready-for-use Powervision peroxidase system (Immunologic, Duiven, The Netherlands) and 3,3′-diaminobenzidine (DAB; Sigma; Immunologic) or 3-amino-9-ethyl carbazole (AEC, Sigma) were used to visualize the antibodies. Sections were counterstained with haematoxylin. Sections incubated without the primary antibody were essentially blank.

## Discussion

Rabies occurs on all continents apart from Antarctica. The causative viruses belong to the family Rhabdoviridae, genus *Lyssavirus* [13]. The genus includes rabies virus, European bat lyssavirus 1 and 2, Australian bat lyssavirus, Lagos bat virus, Mokola virus, and Duvenhage virus. The latter three together with rabies virus are found in Africa. Four more lyssaviruses have been isolated from bats (see [14],[15]). It is estimated that 55,000 persons die of rabies each year, 31,000 in Asia and 24,000 in Africa [16]. In the vast majority of cases, human infection is due to the bite of an infected mammal, mostly dogs. The bite, especially of bats, may be unnoticed [17],[18]. Airborne transmission is possible but extremely rare [19]. Transmission via transplantation of cornea, organs, and a vascular segment from donors with unrecognized rabies has occurred [20],[21]. Apart from these transplant cases, no laboratory-confirmed human-to-human rabies transmission has been documented [22]. Two Ethiopian cases of human-to-human transmission by salivary contact, notably a bite and kissing, have been suggested, but both lack laboratory confirmation of the cause of death [23].

Diagnosis of rabies depends on demonstration of the virus or antibodies to the virus in various specimens (serum, saliva, CSF, a skin biopsy with hair follicles taken from the neck, cornea impression smear, and brain biopsy). Several methods are available: RT-PCR, immunofluorescence test, virus neutralization test, and virus isolation in cell culture or animals. In the early stages of disease, results may be negative and tests may have to be performed several times. Once neurological symptoms occur, death is (almost) universally inevitable. Only one patient, who had not received pre- or postexposure prophylaxis, has survived [1]. This 15-year-old patient was diagnosed with encephalitis. She did not receive rabies immunoglobulin or rabies vaccine. Serum and CSF from day 6 after the start of symptoms contained antibodies to rabies virus but virus was not isolated, viral antigen could not be detected, and attempts to amplify viral nucleic acid were unsuccessful. She was treated with induction of coma with benzodiazepines and barbiturates, ketamine, a substance that had shown specific activity against rabies in animal models [24], and with the antiviral drugs ribavirin and amantadine. Twenty-seven months after the bat exposure there were sequelae of dysarthria and gait difficulties, but she had graduated from high school [2]. It was suggested that infection by an attenuated variant bat rabies virus or early presence of rabies antibodies in serum and CSF could have contributed to the success [1],[25],[26].

We applied the same methods of coma induction with benzodiazepine and phenobarbital with continuous monitoring of bursts on the EEG and administered ketamine, ribavirin, and amantadine as reported by Willoughby et al. [1]. Unlike the surviving patient [1], our patient was given rabies vaccine and HRIG on admission on the advice of the NCCID, as at admission the diagnosis of rabies was not evident, though possible, and a differential diagnosis had to be considered. It has been suggested, but it is not definitely proved, that administration of vaccine and immunoglobulin may be harmful once clinical manifestations of rabies have appeared [26]. Of the 11 patients with rabies who were treated with coma induction, ketamine, and antiviral drugs and who did not survive [3], three were not given vaccine or immunoglobulin [4],[5], two received both vaccine and HRIG [6],[7], and one HRIG only and no vaccine [8]; of five mentioned by Wilde et al. [3] no details could be found. In 2003, experts advised that rabies vaccine, immunoglobulin, ribavirin, interferon alpha, and ketamine, preferably in combinations, be considered for the management of rabies [27], advice that was not refuted in later publications [25],[28]. Recently, a Brazilian survivor of rabies was reported on ProMED-mail ([29]; see also [30] for questions and answers). This patient differs from our and other non-surviving patients in several aspects. This 15-year-old boy was attacked by a bat. He received four doses of rabies vaccine without immunoglobulin before becoming symptomatic. The first dose was given 4 days after the bat bite. Neutralizing antibodies were present in serum and CSF, probably early in the disease, but the exact timing is not given. Virus RNA was not detected in CSF. He was treated with midazolam, ketamine, and amantadine and survived. As mentioned, this patient differs from the non-surviving patients because of the early administration of vaccine and presence of antibodies. Details and follow-up results are awaited.

Rabies remains a virtually always fatal disease. In a patient with symptomatic rabies with neutralizing antibodies in the CSF early in the disease, an attempt at intensive treatment inclusive of respiratory and cardiovascular support may be successful. Otherwise, as there are no new data from in vitro and animal studies and no advice from experts on combination treatments, the best possible medical and nursing care with relief of symptoms and comfort care of the patient and support of the family is probably what can be offered at present [3]. Studies of treatment in animal models are urgently needed.

This is the third human case of rabies due to Duvenhage virus infection. This virus was for the first time isolated in 1970 from a human patient in South Africa [31]. Paweska et al. reported on the second South African patient in 2006 and referred to literature on isolations of the virus from bats in South Africa and Zimbabwe [32]. It was not known to be present in Kenya, where rabies is known to occur in dogs and a variety of animals [33]–[35], but until recently not in bats. Results of the first bat survey for lyssaviruses in Kenya, performed in 2006 and 2007, were published in 2008; Lagos bat lyssavirus was found [36]. The camp wardens and health personnel in the area where our patient was infected were not aware of the risk of bat rabies.

This tragic incident is another reminder that even after a minor animal-related injury the wound should be washed thoroughly and postexposure prophylaxis should be given. Where limited access to postexposure prophylaxis may be expected, pre-exposure prophylaxis by vaccination is advised for travellers, especially when travelling with children for longer periods. Human diploid cell vaccine protects against classic rabies virus strains and Duvenhage virus, but not Mokola virus and Lagos bat virus; Duvenhage virus is neutralized by RIG, but Mokola and Lagos bat viruses are not [37],[38].
